# Reduced Systemic Levels of Bile Acids in Individuals with Coronary Artery Disease: Insights from a Systematic Review

**DOI:** 10.3390/ijms26188764

**Published:** 2025-09-09

**Authors:** Víctor Manuel López Espinosa, Francisco J. Amaro-Gahete, Francisco J. Osuna-Prieto

**Affiliations:** 1 Cardiology Department, University Hospital Virgen de las Nieves, 18014 Granada, Spain; lopezvictorcar@gmail.com; 2 Department of Physiology, Faculty of Medicine, Sport and Health University Research Institute (iMUDS), University of Granada, 18010 Granada, Spain; amarof@ugr.es; 3 CIBER de Fisiopatologia de la Obesidad y Nutricion (CIBEROBN), Instituto de Salud Carlos III, 28220 Madrid, Spain; 4 Biosanitary Research Institute, Ibs.Granada, 18012 Granada, Spain; 5 Hospital Universitario Joan XXIII de Tarragona, Institut d’Investigació Sanitària Pere Virgili (IISPV), 43005 Tarragona, Spain; 6 CIBER de Diabetes y Enfermedades Metabólicas Asociadas (CIBERDEM), Instituto de Salud Carlos III (ISCIII), 28029 Madrid, Spain

**Keywords:** atherosclerosis, lipid metabolism, cardiovascular risk, biomarkers

## Abstract

Bile acids (BAs) play a key role in cholesterol metabolism and inflammation. Although altered circulating BA profiles have been reported in cardiometabolic disorders such as type 2 diabetes (T2D) and obesity, their relationship with coronary artery disease (CAD) remains poorly understood. We conducted a systematic review of human studies searching PubMed, Web of Science, and Scopus, assessing circulating BA concentrations in adults with angiographically confirmed CAD compared to non-CAD (NCAD) controls. Risk of bias was evaluated using the Newcastle–Ottawa Scale. From 2782 records, four observational studies met the inclusion criteria. All reported lower circulating BA concentrations in individuals with CAD compared to NCAD controls, with differences ranging from −5.4% to −52.8%. Two studies found a significant inverse association between BA levels and CAD. One study reported lower BA levels only in CAD in men, while another found the reduction more pronounced in individuals with T2D. However, all studies were observational, and most lacked adjustment for confounders such as sex and age. Current evidence suggests that lower circulating BA levels are linked to CAD and may be influenced by sex and T2D status. Further mechanistic and prospective studies are needed to clarify the relevance and directionality of this association.

## 1. Introduction

Coronary artery disease (CAD) remains the leading cause of death worldwide [1]. A key clinical challenge lies in identifying circulating biomarkers that provide valuable insights into the presence, severity, and prognosis of CAD [2]. Although conventional biomarkers such as natriuretic peptides, glycated hemoglobin, lipoprotein subfractions (e.g., LDL-C), and inflammatory cytokines are widely used in clinical practice, their diagnostic and prognostic utility is limited by low sensitivity, substantial interindividual variability, and often insufficient specificity for the complex metabolic networks underlying atherogenesis and plaque progression [3,4,5].

In this context, bile acids (BAs) have emerged as mechanistically relevant yet underexplored biomarkers with potential diagnostic and therapeutic value in cardiometabolic and vascular diseases [6]. Primary BAs, synthesized by hepatocytes, represent the main pathway for cholesterol elimination [6]. Once secreted into the intestinal lumen, they undergo microbiota-mediated biotransformation into secondary BAs, contributing to the structural and functional diversity of the circulating BA pool [7]. Long recognized for their role in facilitating dietary lipid absorption, BAs also reach systemic circulation and function as endocrine signaling molecules. By engaging various nuclear and membrane-bound receptors (i.e., Farnesoid X Receptor (FXR), Pregnane X Receptor (PXR), Vitamin D Receptor (VDR), and G protein-coupled bile acid receptor 1 (TGR5)), BAs modulate key physiological pathways involved in glucose metabolism, vascular tone, and inflammation [8].

Emerging evidence suggests that circulating BAs may serve as biomarkers in CAD [9]. As regulators of lipid and glucose homeostasis, systemic inflammation, and vascular function, BAs intersect with key biological pathways involved in atherogenesis [9]. Furthermore, alterations in BA composition and concentration have been consistently observed in metabolic disorders such as type 2 diabetes (T2D) and dyslipidemia, both of which are well-established risk factors for CAD [10]. In addition, BAs exert immunomodulatory effects, influence endothelial barrier integrity, and shape gut microbial ecology, factors increasingly implicated in cardiovascular disease pathophysiology [11].

However, despite growing evidence linking BAs and CAD, no comprehensive systematic review has been conducted to date. Therefore, we aimed to systematically review and critically evaluate the relationship between circulating BAs and CAD in humans, with particular emphasis on the potential mechanisms underlying BA involvement in CAD pathogenesis.

## 2. Materials and Methods

This work was conducted in accordance with the PRISMA guidelines for systematic reviews to ensure transparency and reproducibility. Eligibility criteria were determined based on the PICO (Participants, Intervention, Comparison, and Outcome) framework [12]. The review protocol was registered in PROSPERO (CRD420251000102) to uphold methodological rigor and transparency.

### 2.1. Search Strategy

**Figure 1:**
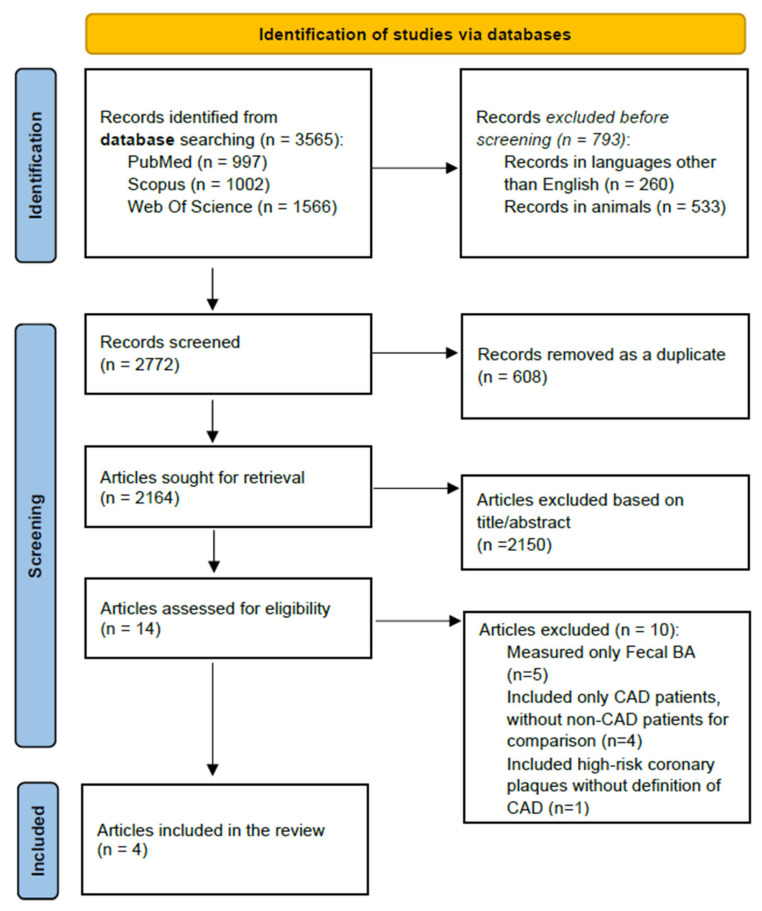
PRISMA 2020 flow diagram illustrating the study selection process.

A comprehensive systematic search was conducted across three electronic databases: PubMed (National Center for Biotechnology Information, Bethesda, MD, USA), Web of Science (Clarivate Analytics, Philadelphia, PA, USA), and Scopus (Elsevier, Amsterdam, The Netherlands), in November 2024 (see Figure 1). The search strategy combined controlled vocabulary (Medical Subject Headings, MeSH) and free-text terms to identify studies investigating the association between circulating bile acids and coronary artery disease. The MeSH terms included “Bile Acids and Salts”, “Coronary Artery Disease”, “Myocardial Infarction”, “Acute Coronary Syndrome”, and “Angina, Unstable”. Additional keywords were incorporated to maximize sensitivity: “serum bile acid*”, “plasma bile acid*”, “coronary*”, “ST-Elevation Myocardial Infarction”, “Non-ST-Elevation Myocardial Infarction”, and “unstable angina”. The search algorithm used in PubMed was (bile acid* [Title/Abstract] OR bile* [Title/Abstract] OR TBA [Title/Abstract]) AND (coronary [Title/Abstract] OR CAD [Title/Abstract] OR STEMI [Title/Abstract] OR NSTEMI [Title/Abstract] OR unstable angina [Title/Abstract]). In Web of Science, the following query was used: TS = (“bile acid*” OR “bile*” OR “TBA”) AND (“coronary” OR “CAD” OR “STEMI” OR “NSTEMI” OR “unstable angina”). In Scopus, the search string was TITLE-ABS (“bile acid*” OR “bile*” OR “TBA”) AND TITLE-ABS (“coronary*” OR “CAD” OR “STEMI” OR “NSTEMI” OR “unstable angina”). Filters for human studies and English-language publications were applied in all databases (except for the “humans” filter in Web of Science, which was not available). Duplicate records (*n* = 624) were removed during the initial screening using EndNote X9 (Clarivate Analytics, Philadelphia, PA, USA).

### 2.2. Inclusion Criteria

Studies involving adults with or without CAD were included. Included studies had to measure circulating BAs in serum or plasma using High-Performance Liquid Chromatography–Tandem Mass Spectrometry (HPLC-MS/MS), enzyme-linked immunosorbent assay (ELISA), or Total Bile Acids (TBAs) assay kit-based techniques. Only original research articles from clinical trials, cohort studies, case–control studies, or cross-sectional studies published in English were considered.

### 2.3. Exclusion Criteria

We excluded manuscripts that determined BA only in feces, as well as those conducted in animals, pregnant women, and children. This exclusion was implemented to ensure methodological consistency and clinical relevance in humans. Circulating levels of BA are a better proxy of systemic metabolism, including synthesis, enterohepatic circulation, and elimination [13,14]. By contrast, local factors such as microbiota, diet, and intestinal motility influence fecal BA levels, introducing variability that complicates comparisons. Since the circulating BA pool is regulated by FXR, which controls BA synthesis through factors like fibroblast growth factor 19, an inhibitor of 7 alpha-hydroxylase (CYP7A1), and the key enzyme in hepatic bile acid production [15], the BA pool in the intestinal tract may not directly reflect their systemic effects or their association with CAD. This is particularly relevant given the presence of BA receptors in cardiac muscle, brown adipose tissue, blood vessels, and the brain [16], which suggests broader physiological roles beyond the gastrointestinal system.

Narrative reviews, guidelines, protocols, letters, conference abstracts, unpublished studies, case reports, and case series were also excluded.

### 2.4. Participants

Adults (>18 years old) with CAD and those without CAD were included in the systematic review. We evaluated CAD using either coronary angiography or computed tomography.

### 2.5. Comparisons

We selected studies that measured circulating TBA levels in plasma or serum and compared them between patients with CAD vs. non-CAD (NCAD) controls.

### 2.6. Outcome (Assessment)

Differences in circulating BA levels between CAD and NCAD patients, and associations between BA levels and CAD presence.

### 2.7. Study Identification and Selection

Two independent researchers screened the studies based on the inclusion and exclusion criteria. Any disagreements were resolved by consensus or by consulting a third independent researcher. Full-text articles were retrieved for final assessment. The following data were extracted from the included publications: year of publication, participant characteristics (i.e., age, sex, T2D), presence or absence of CAD, and circulating BA concentration (serum or plasma) measured by HPLC-MS/MS, ELISA, or TBA enzymatic kit (see Table 1). Descriptive BA levels were retrieved from the studies included. In total, four studies fulfilled all eligibility criteria and were included in the qualitative synthesis. Ten additional studies were excluded after full-text review because they measured only fecal BAs (*n* = 5), including only CAD patients without an NCAD comparison group (*n* = 4) or focused on high-risk coronary plaques without a clear definition of CAD (*n* = 1). Some of them are nevertheless cited in the Discussion section to provide further context [17,18,19]. A quantitative meta-analysis was not conducted due to the limited number of eligible studies finally (*n* = 4) and considerable methodological heterogeneity. Methodological quality was independently assessed by two researchers using the Newcastle–Ottawa Scale (NOS, Table 2) [20].

## 3. Results

The flow diagram (Figure 1) illustrates the study selection process. A total of 3565 studies were initially identified through PubMed, Scopus, and Web of Science. Of these, 793 were excluded for being non-English publications (*n* = 260) or animal studies (*n* = 533). After removing 608 duplicates, 2164 studies remained for screening. Following title and abstract review, 2150 studies were excluded due to irrelevance to the research question, inadequate study design, absence of primary data, or lack of evaluation of circulating BAs. Fourteen full-text articles were assessed for eligibility; among these, one focused on high-risk coronary plaques without CAD classification by computed tomography, nine measured fecal bile acids, and four included only CAD patients without a control group. Ultimately, four observational, cross-sectional studies [21,22,23,24] met the inclusion criteria and were included in the present systematic review.

Table 1 shows the key methodological, clinical features, and findings of the included studies. Overall, all studies (*n* = 4) consistently report reduced circulating TBA levels [21,22,23] or specific BA species concentrations [24] in patients with CAD compared to NCAD controls. Feng et al. [23] analyzed 20,255 postmenopausal women, including 6421 with T2D, and found serum TBA levels were 5.4% lower in patients with CAD or MI, and 10.3% lower in those with both CAD and T2D. CAD menopausal women were older than NCAD. Low TBA remained independently associated with increased CAD risk in the T2D subgroup. Furthermore, Nguyen et al. [22] studied 80 patients undergoing coronary angiography and reported ~53% lower serum TBA levels in those with CAD in comparison to NCAD patients. Despite CAD patients being older than NCAD, reductions extended across primary, secondary, and conjugated BAs, and low TBA levels remained independently associated with CAD after adjustment for age and sex. Notably, in a subgroup analysis, statin therapy was associated with a two-fold increase in TBA levels. Supporting these findings, in a cohort of 7438 patients undergoing coronary angiography, Li et al. [21] reported significantly lower circulating TBA levels in individuals with CAD compared to NCAD, with further reductions observed in cases of myocardial infarction (MI). TBA concentrations below the median (3.6 μmol/L) were independently associated with a two-fold increased risk of CAD and MI, highlighting their potential utility in cardiovascular risk stratification. Importantly, CAD patients were older than NCAD. Finally, Bay et al. [24] explored sex-specific differences in a cohort of 177 patients. Sex-stratified analyses showed that CAD in men, but not in women, was associated with reduced levels of secondary BAs glycolithocholic acid (GCLA) and lithocholic acid (LCA). In this study, CAD patients were also older than NCAD controls.

## 4. Discussion

To our knowledge, this is the first systematic analysis of the relationship between circulating BAs and CAD in humans. Across all four included studies, BA levels were consistently lower in patients with CAD compared to NCAD controls, with reductions ranging from −5.4% to −52.8%. Two large cohort studies demonstrated that low BA concentrations were independently associated with the presence of CAD and MI; notably, one of these studies reported more pronounced reductions among individuals with T2D. In a sex-stratified study analysis, CAD in men, but not in women, was associated with reduced levels of secondary BAs, suggesting the presence of sex-specific metabolic signatures. Collectively, these findings highlight low circulating BA as a reproducible metabolic feature of CAD and MI, supporting a putative role for BA metabolism in disease pathogenesis.

Building on this evidence, several studies have also examined the prognostic significance of systemic BA concentrations, linking reduced levels not only to CAD incidence but also to MI and the severity of coronary lesions. Two of the studies included in this review showed that systemic BA levels are associated with CAD incidence, MI, and the severity of coronary lesions [21,22]. Supporting this, Liu et al. [17] found that lower TBA levels were closely linked to the severity of coronary lesions and increased mortality in patients with acute coronary syndrome (ACS), highlighting their potential prognostic value in predicting adverse cardiovascular events. Another study reported that ACS patients who had fasting TBA levels ≤ 3.1 µmol/L had a markedly elevated risk of major adverse cardiovascular events two years after percutaneous coronary intervention [18]. Furthermore, Zeng et al. [19] reported that patients with moderately low TBA levels (3.5–10 μmol/L) had a decreased risk of major adverse cardiovascular events in comparison to those with TBA levels ≤ 3.5 μmol/L. Collectively, these findings suggest that systemic BA levels may serve as valuable biomarkers for the presence and severity of CAD. However, the current evidence remains limited to observational, cross-sectional studies.

The interplay between BA metabolism, T2D, and CAD represents an additional layer of complexity, as alterations in glucose homeostasis may further modulate BA profiles and their vascular effects. Feng et al. [23], for instance, reported a negative linear association between TBA levels and CAD, particularly among patients with T2D. This finding supports the idea that T2D may amplify the reduction in circulating BAs observed in CAD. One possible contributing factor is insulin, which plays a key regulatory role in hepatic BA synthesis by inhibiting enzymes such as CYP7A1 and sterol 27-hydroxylase (CYP27A1), both of which catalyze rate-limiting steps in BA production [25]. Furthermore, BAs also modulate glucose homeostasis. FXR activation, for instance, decreases hepatic glycolysis and slows intestinal glucose absorption, favoring glycogen storage over glycolysis [26]. TGR5, conversely, augments insulin secretion and β-cell functionality through glucagon-like peptide 1 (GLP-1) stimulation, thereby connecting BA signaling to pancreatic function and glucose regulation [27]. Interestingly, intestinal FXR inactivation (via BA sequestrants, genetic deficiency, or alteration in gut microbiota) has been shown to enhance glucose and energy balance, illustrating the nuanced and context-dependent influences of BAs on metabolic pathways [28]. While some studies reported an increase in circulating TBA levels in diabetic individuals [29], others did not observe significant changes [30]. However, changes in total TBA levels do not reflect individual differences in BA species. For example, diabetic patients show higher plasma levels of deoxycholic acid (DCA) relative to CDCA, whereas non-diabetic individuals tend to have higher levels of cholic acid (CA) [31]. Therefore, a species-level analysis of circulating BAs is essential to elucidate their distinct contributions to CAD pathogenesis.

To place these results in context, we next discuss potential mechanisms linking reduced circulating BA levels to CAD pathophysiology. BAs play a key role in regulating lipid and glucose metabolism, inflammation, and cholesterol homeostasis, all of which are critical processes in the development of CAD [32]. Several studies have shown that reduced BA production can lead to the accumulation of cholesterol, contributing to the pathophysiology of atherosclerosis [33]. This hypothesis is supported by the fact that BA-sequestering agents (e.g., cholestyramine) lower LDL cholesterol by inhibiting BA reabsorption in the intestine. This process prompts the liver to convert cholesterol into BAs, which in turn lowers circulating cholesterol levels [34]. Thus, impaired cholesterol-to-BA conversion may lead to increased circulating cholesterol levels, a fact that could drive the progression of CAD [35]. Beyond cholesterol metabolism, the reduced BA levels in humans might impair FXR and TGR5 signaling, thereby enhancing NF-κB–mediated inflammation in macrophages and vascular cells, exacerbating cholesterol accumulation and endothelial dysfunction. Chenodeoxycholic acid (CDCA), for instance, acts as a dose-dependent inhibitor of pro-inflammatory cytokines such as interleukin-1, interleukin-6, and TNF-α [36,37]. Dual activation of FXR and TGR5 might reduce atherosclerosis mainly through anti-inflammatory rather than lipid-lowering effects [38]. FXR activation suppresses NF-κB signaling and cytokine expression in vascular and immune cells, whereas FXR deficiency accelerates inflammation and plaque development in animal models [38,39]. On the other hand, TGR5, a membrane G protein-coupled BA receptor, is expressed in macrophages, endothelial cells, and other immune cells implicated in atherogenesis. Activation of TGR5 inhibits NF-κB–dependent pro-inflammatory signaling [40,41,42]. This suppresses macrophage uptake of oxidized LDL and reduces production of pro-inflammatory cytokines, thereby attenuating the chronic vascular inflammation central to plaque formation and instability [40,41,42]. Altogether, findings from preclinical studies suggest that reduced circulating BA levels may diminish FXR/TGR5-mediated anti-inflammatory protection, creating a permissive environment for vascular inflammation, plaque formation, and ultimately CAD progression.

Last, but not least, it is important to consider that pharmacological interventions such as statins further complicate the interpretation of BA profiles in CAD. Statins influence systemic BA levels primarily through complex regulatory effects on BA synthesis and transport, with important implications for CAD. In mice treated with atorvastatin, statins increase the expression of CYP7A1, the rate-limiting enzyme in BA synthesis, by repressing FXR signaling in the liver and intestine [43]. Consequently, the increment of CYP7A1 expression would compensate for reduced cholesterol synthesis, thus maintaining BA homeostasis [43]. Among the studies that registered statin usage [21,23,24], two of them reported higher statin use in the CAD patients [21,23]. Furthermore, in a subgroup of 17 patients, Nguyen et al. showed that statin therapy doubled the serum BA concentration [22]. Thereby, although statin therapy is likely to increase systemic BA levels, the majority of NCAD participants were also receiving statins, and BA levels were still lower in the CAD group. The interaction between statins and BA metabolism in CAD patients, specifically among those with T2D, warrants further investigation, and future studies should consider this type of medication when analyzing the BA levels and CAD.

### Limitations of the Studies Included in the Systematic Review

Several limitations restrict the reliability and generalizability of the results of the studies included in the present review. First, variability in BA measurement techniques, which varied from less specific enzymatic assays [21,23] to highly sensitive, such as HPLC-MS/MS [22,24], precludes cross-study comparisons. This is clinically relevant since accumulating evidence suggests that different BA subtypes may play distinct roles in cardiovascular health [31]. Second, two studies exclusively examined Chinese populations [21,23], whereas the rest focused on Western European individuals [22,24], limiting the conclusions due to potential ethnic differences in BA metabolism and cardiovascular risk. Future studies should include diverse populations, as ethnicity influences systemic lipid metabolism, BA levels, and cardiovascular risk through genetic, metabolic, and microbial differences [32,44]. Regarding statistical analyses and confounders, the study of Li et al. [21] included participants with T2D without appropriate statistical adjustments, potentially introducing bias. Sex is a potential confounder in BA profiles, as shown by Li et al. [22], who found differences in BA levels in men but not in women, while sex-related hormonal differences may further modulate BA composition and circulation, an effect supported by multiple studies demonstrating sex-specific BA metabolism influenced by hormonal, genetic, and microbiota-related factors [45]. Aging is another factor associated with metabolic changes that could affect BA regulation [46]. While Bay et al. [24] matched the analysis, including sex, the rest of the studies did not account for this factor, even though CAD patients were significantly older than their NCAD counterparts [21,22,23]. Finally, the lack of longitudinal follow-up in the studies prevents the establishment of causality. Therefore, while associations between BA levels and CAD have been observed, it remains unclear whether these changes contribute to disease development or are a consequence of it. Future studies should consider these limitations in their design and analysis to provide unbiased and more conclusive results regarding the relationship between systemic BA levels and CAD in humans.

## 5. Conclusions

Our findings indicate that lower circulating BA levels are associated with the presence of CAD in humans. This association may reflect impaired cholesterol-to-BA conversion or disruptions in enterohepatic circulation, which are potential mechanisms contributing to CAD pathophysiology. Further research is warranted to clarify the causal pathways and mechanistic roles of BAs in CAD.

## Figures and Tables

**Table 1:** Characteristics and findings of the studies comparing circulating bile acid levels in individuals with and without coronary artery disease.

Reference	Study Design	Aim	BA Measurement	Country	Statins Use (%)	Participants	Results
Li et al., 2019. [21]	Observational, cross-sectional	Investigate the association between TBA levels and the incidence of CAD, MI, and coronary lesions	TBA enzymatic assay	China	CAD = 97.8% NCAD = 94.5%.	CAD (*n* = 5853; 62 ± 10 yr.; 28% female), NCAD (*n* = 1585; 60 ± 10 yr.; 49% female)	Patients with CAD had 15% lower serum TBA levels than those with NCAD (3.4 vs. 4 µmol/L). Lower TBA levels were associated with CAD, MI, and the severity of coronary lesions in patients with suspected CAD
Nguyen et al., 2021. [22]	Observational, cross-sectional	Compare BA levels of CAD and NCAD patients	HPLC-MS/MS	France	NR	CAD (*n* = 45; 66 ± 1 yr.; 27% female) non-CAD (*n* = 35; 57 ± 2 yr.; 51% female)	Patients with CAD presented 53% lower serum TBA levels than those without NCAD. These differences were also significant for primary, secondary, and conjugate BAs, and glycochenodeoxycholic acid
Feng et al., 2021. [23]	Observational, cross-sectional	Explore the relationship between TBA levels and MI or CAD, with or without T2DM, in menopausal women	TBA enzymatic assay	China	CAD = 83.3% NCAD = 69.7%	CAD (*n* = 12,639; 65 [60–70] yr.) NCAD (*n* = 7616; 63 [58–68] yr.) CAD + T2D (*n* = 4701) NCAD + T2D (*n* = 1720) All patients were menopausal females	Women with CAD had 5.4% lower serum TBA levels than NCAD. Women with CAD + T2D had 10.3% lower levels than NCAD + T2D Lower TBA also correlated with MI in groups with and without T2D.
Bay et al., 2024. [24]	Observational, cross-sectional	Investigate the lipidomic and BA profile differences between CAD and NCAD patients	HPLC-MS/MS	Germany	CAD men = 20.5% CAD women = 27.3% NCAD men = 15.6% NCAD women = 22.7%	CAD (*n* = 88; 72 ± 2 yr.; 51% female) NCAD (*n* = 89; 68 ± 13 yr.; 51% female)	Men, but not women, CAD patients presented lower secondary glycolithocholic and lithocholic BA levels vs. NCAD patients

Data are presented as mean ± standard deviation (SD) or [range]. BA: Bile Acids, CAD: Coronary Artery Disease, HPLC-MS/MS: High-Performance Liquid Chromatography–Tandem Mass Spectrometry, MI: Myocardial Infarction, NCAD: Non-Coronary Artery Disease, NR: Not Reported, T2D: Type 2 Diabetes Mellitus, TBA: Total Bile Acids, yr: years.

**Table 2:** Newcastle–Ottawa Scale (NOS) quality assessment of the included studies.

First Author, Year	Selection (0–4)	Comparability (0–2)	Outcome (0–3)	Total Score (0–9)	Quality
Li et al., 2019. [21]	★★★★	★★	★★	8	High
Nguyen et al., 2021. [22]	★★★	★	★★	6	Moderate
Feng et al., 2021. [23]	★★★★	★★	★★★	9	High
Bay et al., 2024. [24]	★★★	★	★★	6	Moderate

The NOS evaluates three domains: Selection (maximum 4 stars), Comparability (maximum 2 stars), and Outcome/Exposure (maximum 3 stars). Scores range from 0 to 9, with higher scores indicating better methodological quality (0–3 = low, 4–6 = moderate, 7–9 = high). Each star (★) represents one point awarded when the corresponding methodological criterion was fulfilled in that NOS domain.

## Data Availability

The original contributions presented in this study are included in the article. Further inquiries can be directed to the corresponding author.

## Abbreviations

The following abbreviations are used in this manuscript:

ACS	Acute Coronary Syndrome
BA	Bile Acid
BMI	Body Mass Index
CAD	Coronary Artery Disease
CYP27A1	Sterol 27-Hydroxylase
CYP7A1	Cholesterol 7α-Hydroxylase
DCA	Deoxycholic Acid
DM	Diabetes Mellitus
ELISA	Enzyme-Linked Immunosorbent Assay
FMO3	Flavin-Containing Monooxygenase 3
FXR	Farnesoid X Receptor
GLCA	Glycolithocholic Acid
GLP-1	Glucagon-Like Peptide-1
HPLC-MS/MS	High-Performance Liquid Chromatography–Tandem Mass Spectrometry
LCA	Lithocholic acid
LDL	Low-Density Lipoprotein
LPS	Lipopolysaccharides
MI	Myocardial Infarction
NCAD	Non-Coronary Artery Disease
NOS	Newcastle–Ottawa Scale
NR	Not Reported
OR	Odds Ratio
PXR	Pregnane X Receptor
T2D	Type 2 Diabetes
TBA	Total Bile Acids
TGR5	Takeda G Protein-Coupled Receptor 5
TMA	Trimethylamine
TMAO	Trimethylamine N-oxide
VDR	Vitamin D Receptor
